# Ataxia Rating Scales Reflect Patient Experience: an Examination of the Relationship Between Clinician Assessments of Cerebellar Ataxia and Patient-Reported Outcomes

**DOI:** 10.1007/s12311-022-01494-1

**Published:** 2022-12-10

**Authors:** Michele H. Potashman, Miranda L. Mize, Melissa W. Beiner, Samantha Pierce, Vladimir Coric, Jeremy D. Schmahmann

**Affiliations:** 1 Biohaven Pharmaceuticals, New Haven, CT, USA; 2 Ataxia Center, Cognitive Behavioral Neurology Unit, Laboratory for Neuroanatomy and Cerebellar Neurobiology, Massachusetts General Hospital and Harvard Medical School, Boston, MA USA; 3 Department of Neurology, Massachusetts General Hospital, 100 Cambridge Street, Suite 2000, Boston, MA 02114 USA

**Keywords:** Clinical outcome assessments, Clinician-rated measures, ClinROs, Ataxia rating scales, Patient Reported Outcome Measure of Ataxia (PROM-Ataxia), Concept elicitation

## Abstract

Ataxia rating scales are observer administered clinical outcome assessments (COAs) of the cerebellar motor syndrome. It is not known whether these COAs mirror patient experience of their disease. Here we test the hypothesis that ataxia COAs are related to and reflect patient reported symptoms and impact of illness. A concept library of symptoms and activities impacted by ataxia was created by reviewing (a) concept elicitation data from surveys completed by 147 ataxia patients and 80 family members and (b) cognitive debrief data from focus groups of 17 ataxia patients used to develop the Patient Reported Outcome Measure of Ataxia. These findings were mapped across the items on 4 clinical measures of ataxia (SARA, BARS, ICARS and FARS). Symptoms reported most commonly related to balance, gait or walking, speech, tremor and involuntary movements, and vision impairment. Symptoms reported less frequently related to hand coordination, loss of muscle control, dizziness and vertigo, muscle discomfort or pain, swallowing, and incontinence. There was a mosaic mapping of items in the observer-derived ataxia COAs with the subjective reports by ataxia patients/families of the relevance of these items to their daily lives. Most COA item mapped onto multiple real-life manifestations; and most of the real-life impact of disease mapped onto multiple COA items. The 4 common ataxia COAs reflect patient reported symptoms and impact of illness. These results validate the relevance of the COAs to patients’ lives and underscore the inadvisability of singling out any one COA item to represent the totality of the patient experience.

Disorders of the cerebellum produce the triad of clinical ataxiology—the cerebellar motor syndrome, cerebellar vestibular syndrome, and the cerebellar cognitive affective syndrome [1, 2]. Cerebellar diseases include hereditary forms of ataxia such as spinocerebellar ataxia (SCA) and Friedreich’s ataxia, neurodegenerative disorders in which cerebellar ataxia is a dominant feature (e.g., multiple system atrophy cerebellar type (MSA-C)) [3], and acquired forms of ataxia following focal injuries such as stroke, tumor, and immune-mediated diseases. The details of symptom manifestations, disease progression, and life expectancy vary across the different cerebellar ataxias, but all share the common features of the cerebellar motor syndrome characterized by impaired gait, coordination of voluntary movements, speech articulation, and oculomotor control [4–7]. In neurodegenerative ataxias, symptoms impair activities of daily living (ADLs), impact quality of life for both the patient and family, increase patient reliance on caregivers, and contribute to increased mortality risk [8–13].

Clinical outcome assessments (COAs) are often utilized in neurodegenerative diseases that aim to evaluate a patient’s health status, particularly how a patient functions and feels. The key concepts and activities measured in these disorder-specific scales are often determined based on clinician experience and it remains to be shown whether clinical perception aligns with patient-reported experience of their own disease. This is particularly important for clinician-rated measures (termed “ClinROs”) because demonstrating patient relevance of COAs based on patient-reported data has the potential to increase confidence in the instruments and deepen understanding of the linkage between the clinician-tested measures and patient-experienced symptoms, thereby enhancing face validity of these tools [14–19].

Within the field of ataxia, there is interest in exploring the patient-relevance of a series of clinician-created COA measures commonly used in clinical practice and in research studies of adult patients with progressive cerebellar ataxias. Here, we test the hypothesis that these ataxia rating scales meaningfully reflect disabilities in the cerebellar ataxia patient population. To achieve this aim, we studied the relationship between 4 commonly used clinician-rated assessments and patient-reported experiences in cohorts of patients with cerebellar ataxia.

## Methods

**Fig. 1:**
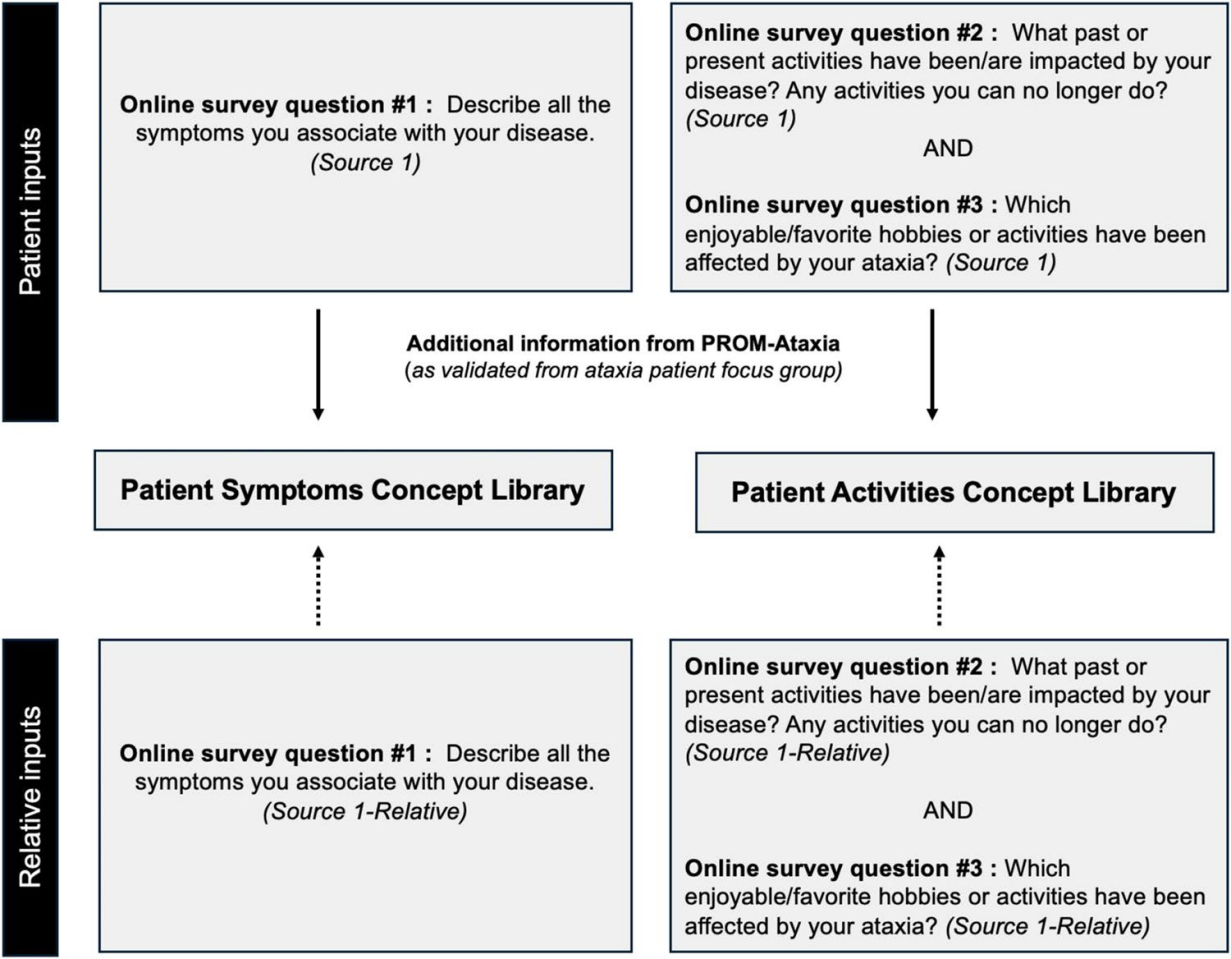
Source inputs for the symptoms and activities concept libraries

In this analysis, we leveraged a mixed-methods approach, evaluating patient data from two sources against a series of concepts evaluated in a group of COAs (Fig. 1) [20]. Source 1 comprised an online concept elicitation survey about patient experience with cerebellar ataxias. The survey was designed to capture symptoms and impacts of ataxia to inform the de novo creation of a patient reported outcome measure of ataxia (PROM-Ataxia); details regarding administration of the survey are described in the original publication of the survey data [21]. In brief, the survey consisted of 6 open-ended questions about the patient experience with ataxia:

1. Describe all the symptoms you associate with your disease.
2. What past or present activities have been/are impacted by your disease? Any activities you can no longer do?
3. Which enjoyable/favorite hobbies or activities have been affected by your ataxia?
4. Can you think of anything in your life that has not been affected by your disease?
5. Do you feel your thinking/emotions have changed since you developed ataxia?
6. What symptoms/affected activities do you feel your physicians may not routinely ask about or do not know about?

Both patients diagnosed with cerebellar ataxia and relatives of someone diagnosed with cerebellar ataxia were invited to complete the survey; language was adjusted in the relatives’ survey to indicate the questions were concerning the patient. A total of 147 patients and 80 relatives completed the survey. Any potential relationship between specific patient respondents and family member respondents was not known. Whereas the analysis focused on the patient-reported data, the responses provided by relatives were also examined to provide additional information.

Responses providing information around the reported symptoms (question 1) and the types of activities patients are unable to do because of their condition (question 2, question 3) were considered the primary data set. The survey responses were provided as free text in sentence form. These were then deconstructed, and the discrete responses identified.

Source 2 is the PROM-Ataxia, in which all items were derived from the concept elicitation step in source 1. The preliminary version of the PROM-Ataxia was subjected to cognitive debrief with a focus group of 17 ataxia patients ([21], led by a research associate and incorporating the guidance of an ataxia expert (JDS). The findings from the survey responses (source 1) and the PROM-Ataxia items (source 2) were collated in an exploratory and iterative process that leveraged thematic analysis methods [22].

Each item on the COAs was organized into higher level health concepts using thematic analysis methods (Table 1). For example, the “health concept” for “gait and walking” reflects different permutations for evaluating this function (e.g., presence of staggering, tandem walking ability, use of personal aids, presence of ataxia symptoms during walking, gait speed). The term “item” is used here to mean each of the specific evaluations or questions captured by the COA (e.g., SARA item #1, gait assessment).

**Table 1:** Concept and item mapping: SARA, FARS, BARS, and ICARS

Concept summarized	Measure and item #	Item details and goals of assessment	Rating Scale range
*Evaluation of gross motor functions*			
Gait and walking	SARA, item #1 BARS, item #1 ICARS, item #1	Evaluates gait, walking, staggering; considers walking aids or personal assistance required; wheelchair bound	0–8^1^
	FARS-ADL, item #7	Evaluates difficulty of walking, imbalance; considers walking aids or personal assistance required; wheelchair bound	0–4^1^
	FARS-Neuro-E, item #7	Evaluates presence of ataxia symptoms during walking; considers walking aids or personal assistance required; wheelchair bound	0–5^1^
	FARS-Neuro-E, item #6	Evaluates ability to walk in tandem, balance; too poorly coordinated to attempt task	0–3^2^
	ICARS, item #2	Evaluates gait speed (as observed during ICARS #1)	0–4
	FARS-Neuro-E, item #7 -time	Evaluates gait speed (timed assessment, captured alongside ordinal rating scale for item #7)	N/A (timed)
Stance and balance *(while standing still)*	SARA, item #2	Evaluates foot position and sway when in stance; considers constant or intermittent supports needed; unable to stand	0–6^2^
	FARS-Neuro-E, item #2 FARS-Neuro-E, item #3 FARS-Neuro-E, item #4 FARS-Neuro-E, item #5	Evaluates time able to stand in specified positions *Item 2: feet apart* *Item 3: feet together* *Item 4: tandem stance* *Item 5: dominant foot*	0–4
	ICARS, item #3	(Eyes open) Evaluates foot position and sway when in stance; considers constant or intermittent supports needed; unable to stand	0–6^2^
	ICARS, item #4	(Eyes open) Evaluates foot position distance needed to obtain comfortable stance	0–4
	ICARS, item #5 ICARS, item #6	Evaluates sway with stance; immediate falling *Item 5: Eyes open* *Item 6: Eyes closed*	0–4
Quality of sitting position	SARA, item #3 FARS-ADL, item #8 FARS-Neuro-E, item #1 ICARS, item #7	Sitting stability and sway, level of supports needed; unable to maintain seated position SARA: arms outstretched FARS-ADL: no specification of arm position ICARS, FARS-Neuro: arms folded	0–4^2^
*Evaluation of physical functioning, targeted systems*			
Speech	SARA, item #4	Level of speech impairment based on frequency of difficult to understand words; speech intelligible	0–6^3^
	FARS-ADL, item #1	Level of speech impairment, considers frequency of being asked to repeat statements; speech intelligible	0–4^3^
	FARS-Neuro A, item #4		
	FARS-Instrumental, item #1	Ability to repeat phrase in fixed time period (timed test)	N/A (timed)
	BARS, item #4	Level of speech impairment based on rate/rhythm/clarity of the speech; speech intelligible	0–4^3^
	ICARS, item #15	Level of speech impairment based on rate and fluency of the speech; speech intelligible	0–4^3^
	ICARS, item #16	Level of speech impairment based on clarity and slurring of words; speech intelligible	0–4^3^
Swallowing	FARS-ADL, item #2	Records choking frequency; considers if patient modifies or avoids certain foods; require NG tube of gastrostomy feedings	0–4
Bladder function	FARS-ADL, item #9	Records status of bladder function, urgency and incontinence; considers if bladder drugs are used; loss of bladder function or intermittent catherization	0–4
Oculomotor	BARS, item #5	Evaluates presence and degree of oculomotor changes (summary score)	0–2
	ICARS, item #17	Rates the persistence gaze-evoked nystagmus	0–3
	ICARS, item #18	Evaluates the severity of saccadic eye movements	0–2
	ICARS, item #19	Indicates the presence or absence of dysmetria (of the saccade)	0–1
Cough	FARS-Neuro-A, item #3	Records ability to cough	0–2
*Evaluation of upper limb functioning*			
Finger movement and target accuracy	SARA, item #5 FARS-Neuro-B, item #3	Assesses precision of finger meeting target; unable to perform movement	0–4^2^
	BARS, item #3 ICARS, item #10	Smoothness of arm movement during task, assess precision of finger meeting target; unable to perform movement	0–4^2^
Fine motor accuracy	FARS-Neuro-B, item #5	Assesses time and precision of finger tapping each other	0–4^2^
	FARS-Instrumental, Item #1	Time on 9-hole peg board task	N/A (timed)
	ICARS, item #14	Evaluates degree of precision of patient drawing of outlined image; unable to perform (or drawing completely disorganized)	0–4^2^
Hand tremor	SARA, item #6 FARS-Neuro-B, item #2	Evaluates degree of hand/finger tremor during task; unable to perform movement	0–4^2^
	FARS-Neuro-B, item #1	Evaluates degree of hand/finger tremor at rest	0–3^2^
	ICARS, item #11 ICARS, item #12	Evaluates degree of hand/finger tremor Item 11: tremor during task Item 12: tremor at rest	0–4
Altering hand movements	SARA, item #7 FARS-Neuro-B, item #4	Evaluates regularity to pattern of hand movement (alternation of pro- and supinations of the hand); unable to perform movement	SARA: 0–4^2^ FARS: 0–3
	ICARS, item #13	Evaluates speed, form and regularity to pattern of hand movement (alternation of pro- and supinations of the hand); unable to perform movement (or movement completely disorganized)	0–4^2^
*Evaluation of lower limb functioning*^*4*^			
Lower limb control	SARA, item #8 BARS, item #2 ICARS, item #8 ICARS, item #9 FARS-Neuro-C, item #1	Evaluates the control (with or without tremor) during the heel-shin slide task; unable to perform SARA, item 8; FARS-Neuro-C, item #1: evaluates ability to control only BARS, item #2; ICARS, item #8: evaluates control and tremor ICARS, item 9: evaluates tremor at knee separately	0–4^2^
	FARS-Neuro-C, item #2	Evaluates control during the heel-shin tap task; unable to perform	0–4^2^
*Evaluation of activities of daily living (ADLs)*			
Cutting food and feeding self	FARS-ADL, item #3	Reflects speed, clumsiness and assistance needed with cutting food and feeding oneself; unable to perform (needs to be fed)	0–4^2^
Dressing	FARS-ADL, item #4	Reflects speed, modifications used, and assistance required with dressing; helpless with dressing-self	0–4^2^
Personal hygiene	FARS-ADL, item #5	Reflects speed, modifications/devices used, and assistance required with hygiene tasks; fully dependent for hygiene	0–4^2^
Falling	FARS-ADL, item #7	Reflects fall frequency; unable to stand or walk	0–4^2^

^1^High score indicates patient is wheelchair bound. ^2^High score indicates patient is unable to perform movement as described. ^3^High score indicates patient has unintelligible speech. ^4^Lower limb evaluations are related to gait and stance/balance abilities

Conceptual models were created that relate each of the identified health concepts to the sets of patient responses (symptoms or impaired activities). Two investigators contributed to the mapping process (MHP and MLM) to enhance consistency (no formal statistic computed). Response frequency was determined by tabulating the number of mentions and reported as high, medium, or low occurrence within the total of survey respondents. Reports by relatives were included if they provided additional details or perspectives. Reported symptoms and impacts were not treated as mutually exclusive as there are proposed relationships between many of the concepts identified [23–26].

**Fig. 2:**
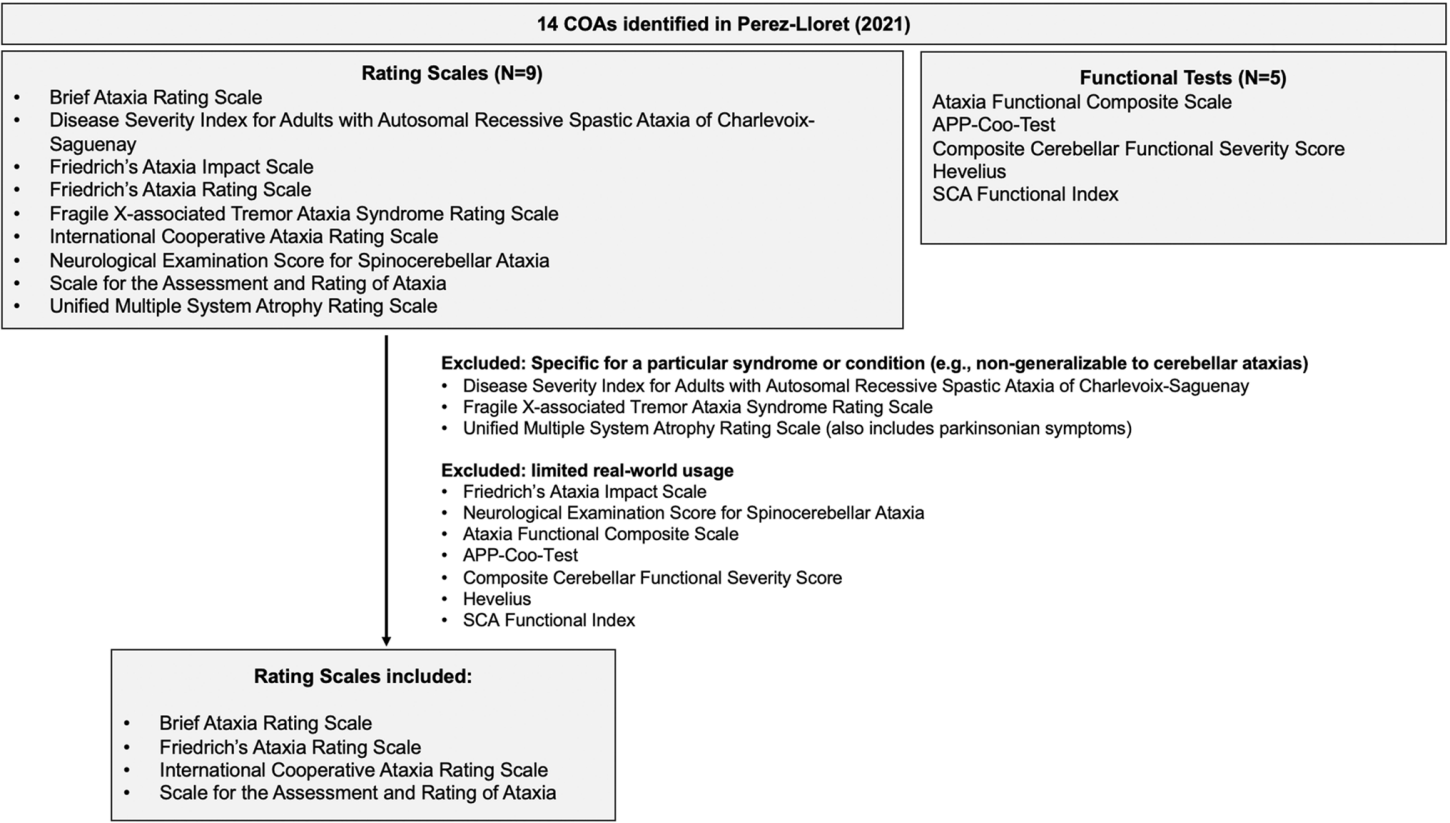
COAs identified in Perez-Lloret SLR evaluating ataxia measures

COAs were selected for inclusion in this study based on their actual or potential use in clinical care and research in persons with cerebellar ataxia. COA measures used to assess ataxia and cerebellar dysfunction were summarized in a recent systematic literature review [27]. Among the 14 COAs identified, 4 were selected for our study, as depicted in Fig. 2. These 4 ataxia scales were deemed to be widely used in clinical practice and/or clinical research by our clinical experts (JDS, MWB) to assess patient symptoms and functional abilities. In addition, consideration was given to be inclusive of measures used in interventional clinical trials (based on postings on clinicaltrials.gov, as of April 2022). Among the measures not selected, 3 targeted specific diseases or syndromes with symptoms beyond the scope of cerebellar ataxia, and 7 are seldom used.

The 4 selected scales are as follows: Scale for the Assessment and Rating of Ataxia (SARA), Friedreich’s Ataxia Rating Scale (FARS), Brief Ataxia Rating Scale (BARS), and the International Cooperative Ataxia Rating Scale (ICARS). Each scale requires a clinician to evaluate signs, symptoms, and/or impacts of cerebellar ataxia and provide a clinical rating score. A description of these measures is as follows.

The SARA assesses ataxia severity and disease progression in clinical practice and research studies. It is available in the public domain [28] and consists of 8 items which evaluate gait and balance, speech, and upper and lower limb coordination [23]. Each item on the SARA is rated on an ordinal scale. The SARA is widely used in patient registries, clinical practice, and interventional studies [29–31]. A modified version was developed for use in the clinical trial setting—the modified functional Scale for the Assessment and Rating of Ataxia (f-SARA) [32].

The FARS is a 4-part assessment consisting of (I) functional staging, (II) the Activities of Daily Living (ADL) scale, (III) the neurological (and symptoms) exam, sections A–E, and (IV) the instrumental assessment [33]. As the functional staging evaluation (domain I) is an overall score of patients’ functioning status, this was deemed too general to be included in this review. It is common to use sub-sections of the FARS that focus on symptoms and activities of daily living [30], and therefore, FARS items that measure only physiologic change (Part III items A1, A2, and section D) were excluded from this review.

The activities of daily living domain (FARS-ADL) (domain II) assesses 9 activities of daily living: speech, swallowing, hand coordination (cutting food and handling utensils), dressing, personal hygiene, falling frequency, walking abilities, quality of sitting position, and bladder function. The FARS neurological and symptoms exam (domain III) comprises sections evaluating bulbar aspects (Section A: cough, speech), upper limb coordination (Section B: tremor with finger to finger, nose-finger; dysmetria with finger chase; rapid alternating hand movements, finger taps), lower limb coordination (Section C: heel-shin slide, heel-shin tap), and upright stability (Section E: sitting posture; stance with feet apart, feet together, tandem, dominant foot; tandem walk, gait). The FARS instrumental assessment (domain IV) is composed of 3 functional tests, the 9-hole peg test, the PATA rate test, and the timed 8-Meter walk. While most items on the FARS are rated using an ordinal scale, the functional tests are performance-based outcomes measures which evaluate the time taken for the prescribed task; the time is typically transformed to a *z*-score using normative values to aid in interpretation. Use of the FARS requires license from the copyright holder.

The BARS was developed as a brief, 5-item assessment of ataxia symptoms [34]. The BARS evaluates gait and balance, upper and lower extremity motor coordination, speech, and oculomotor abnormalities. Each item on the BARS is rated on an ordinal scale. The BARS is available in the public domain and can be found in the original publication [34]. Version 2 of the BARS uses a half-point grading system with each score explicitly detailed. It has been in use since 2013 and has served in studies assessing quantitative measures of ataxia with automated video assessment, computer mouse capture, and extremity and trunk motion sensors [35–40].

The ICARS was one of the earliest measures developed to assess signs and symptoms of cerebellar ataxia [41]. The ICARS consists of 19 items across 5 domains: posture and gait [7 items: walking capacity, gait speed, standing to evaluate balance (eyes open), natural position (eyes open), sway (eyes open and closed), sitting], upper or lower limb coordination [7 items: smoothness of movement with knee-tibia test, tremors with heel to knee and finger to nose test (2 items), decomposition and dysmetria with finger-to-nose, pronation, drawing], speech (2 items: fluency and clarity of speech), and oculomotor disorders (3 items: nystagmus, presence of saccades, and dysmetria). Each item employs an ordinal response scale, with the exception of one item which indicates the presence/absence of saccadic dysmetria. The ICARS is widely used, is available in the public domain, and is found in the original publication [41].

We present our findings in accordance with the consolidated criteria for reporting qualitative studies COREQ checklist [42].

Consent to participate in the online survey was assumed by anonymous participation. All patients participating in the focus group provided written informed consent. Ethical approval and oversight of the online survey and focus group were provided by the institutional review board of Partners HealthCare.

## Results

The items across the 4 COAs were considered indicative of 5 broad categories of physical functioning with 17 distinct health concepts identified (Table 1).

For each concept, the relevant symptoms reported and their frequency are summarized (Table 2). The most frequently reported symptoms were those relating to balance, gait or walking abilities (including falls), speech, tremor and involuntary movements, and vision impairment. Symptoms related to hand coordination, loss of muscle control, dizziness and vertigo, muscle discomfort or pain, swallowing, and incontinence were reported but to a lesser frequency. Generalized symptoms of fatigue and non-specific coordination challenges were also reported.

**Table 2:** Patient-reported symptoms of ataxia: selection of verbatim reports from online survey, question 1 (*N* = 147 patients)

Concept measured	Patient-reported symptoms of ataxia		Frequency of report [high (H, medium (M), low (L)]
*Evaluation of gross motor functions*			
Gait and walking	Walking; gait Tripping; clumsy walking; stumbling; hard to recover from stumble Slow walking; walking as if drunk Cannot turn or stop quickly Wall walking to keep balance; cannot walk without my walker	Cannot walk and carry anything Cannot climb stairs or go down curbs Loss of leg control Random falls weak legs; once in a while my legs will let go on me Falling every week Falling on uneven ground Unable to walk (wheelchair)	H
Stance and balance *(while standing still)*	Balance; difficult regaining balance; balance (feeling of falling backward) Cannot stand *Coordination* *Dizziness*; dizziness particularly when bending over	*My brain feels like it is turning inside my head; vertigo* Vestibular dysfunction Loss of leg control Random falls weak legs; once in a while my legs will let go on me	H
Quality of sitting position	Trunk tremors *Cannot roll over in bed*	Difficulty elevating my body from a sitting position on the floor	L
*Evaluation of physical functioning, targeted systems*^*1*^			
Speech	Speech difficulties; poor speech Occasional speech problems; pronunciation of certain words	Slurred speech; slurred and halting speech Slow speech Difficulty with social conversations	H
Swallowing	Swallowing problems Difficult swallowing pills	Esophageal tremor Choking	M
Bladder function	Urinary incontinence	Bladder weakness; lots of bathroom breaks	L
Oculomotor	Headaches; migraines My brain feels like it is turning inside my head; vertigo Vestibular dysfunction	Dizziness; dizziness particularly when bending over Double vision; blurred vision Lack of depth perception	H
*Evaluation of upper limb functioning*			
Finger movement and target accuracy	Sometimes miss what I am trying to grab; lack of depth perception; over-reaching *Accidentally poking myself in the eye* Double vision; blurred vision	Hand–eye coordination problems; *coordination* *Problems using computer*	M
Fine motor accuracy	Hand coordination; manual dexterity; dexterity challenges; worsening finger dexterity and grasping; problems with fine motor; hard to pick things like coins Writing; cannot write clearly	*Handshakes; tremors; jerkiness; constant movements* Keyboard and typing; poor typing; typing slower *Drop and throwing things around unintentionally*	H
Hand tremor	*Tremors*; handshakes *Jerkiness; twitching;* uncontrollable body movements that make me feel like a puppeteer is in control of my movements (not me)	Constant movements Cannot carry a glass of liquid without spilling *Hand coordination* *Hand/arm clumsiness*	HM
Alternating hand movements	Involuntary movements Problems muscle control; sudden loss of muscle control *Coordination; hand/arm clumsiness* Arm jerk; spasm; twitching	*Manual dexterity; dexterity challenges* *Problems using computer*	H
*Evaluation of lower limb functioning*			
Lower limb assessment	Leg tremors; foot tremors (*see walking and gait responses*)	loss of leg control (*see stance and balance responses*)	H
*Evaluation of activities of daily living (ADLs)*			
Cutting food and feeding self	*Coordination* Hand coordination; hand/arm clumsiness Manual dexterity; loss of grip strength; grip difficulties	Fine motor Handshake Jerkiness; twitching	H
Dressing	*Coordination* Manual dexterity; hard to button small buttons Inability to tie shoes Hard to fasten jewelry *Handshake*	Hand coordination; hand/arm clumsiness Fine motor *Losing grip strength; grip difficulties* *Jerkiness; twitching*	H
Personal hygiene	Worsening dexterity; manual dexterity; fine motor problems; lose grip on small items easily (hairbrush); *can no longer twist on caps* *Balance*	*Coordination challenges*; coordination of hands *Poke myself in eye* *Random falls weak legs; once in a while my legs will let go on me*	MH
Falling	Random falls weak legs; once in a while my legs will let go on me Falling every week	Hard to recover from stumble Falling on uneven ground Falling onto furniture	L

*Italicized responses* reflect reports that may be more tenuously associated with the stated concept, as that the relationship may be less clear. ^1^No symptoms reported reflecting challenges with coughing (e.g., producing a strong, controlled cough)

Symptoms were reported in varying level of detail. As an illustration, within the reports relating to the symptom “balance” were *N* = 95 total responses including “balance” [alone] (*N* = 80) and *N* = 1 each for 15 more detailed responses (such as “balance-at night,” “balance-hard to bike,” “difficult to balance when standing still,” “unstable,” and “very little core balance”).

When considering the relationship of symptoms to the health concepts, some concepts underscored the reported symptoms, such as patient challenges with walking, balance, speech, swallowing, tremors, and urinary incontinence, among others. Several concepts were not as clearly correlated to reported symptoms, and in this case our clinical experts were consulted to best relate these to the symptoms they are designed to assess. The clinical task known as “finger chase,” which involves the patient following a pattern of movement with his or her finger, can be interpreted as an activity to assess symptoms of “hand–eye coordination challenges,” “coordination,” “manual dexterity,” or “lack of depth perception.” The assessment known as “fast alternating hand movements” can be interpreted as assessing symptoms of “coordination,” “dexterity challenges,” “problems muscle control,” “arm jerk,” “spasm,” or “involuntary movements.” Oculomotor impairments tapped several underlying symptoms, such as “double vision,” “blurred vision,” “dizziness,” “vertigo,” “migraines,” and/or “headaches.” Impairment in the act of sitting was not reported overtly as a symptom; however, reports of “trunk tremors,” “can’t roll over in bed,” and “difficulty elevating my body from a sitting position on the floor” possibly indicate the impacts of this core ataxia symptom. The item evaluating cough strength was not a patient-reported concern; however, an increased frequency of coughing was reported as a symptom.

Three of the items on the FARS-ADL were designed to assess the degree of impairment in complex day-to-day activities: cutting food and handling utensils, dressing, and personal hygiene. These three items do not have a singular symptom associated with them, but rather several symptoms that may impact these activities. As an example, the ability to dress one-self may be impacted by symptoms of “coordination challenges,” “losing grip strength,” challenges with “manual dexterity,” “jerkiness,” and/or “balance challenges.” Personal hygiene may be impacted by any combination of symptoms such as “worsening dexterity,” “can no longer twist on caps,” “coordination of the hands,” “loosing grip strength,” “poking myself in the eye,” and/or “random falls weak legs.”

Impacted activities reported by the patient were mapped to each of the health concepts identified across the COAs (Table 3). Similar to the symptom reports, the impacted activities reported varied in specificity. For example, when considering evaluations of gait and walking, “walking” [verbatim] was mentioned numerous times by survey respondents as an impacted activity, and more detailed accounts included responses of “walking longer than 15 min,” “walking while carrying infant,” “walking at night,” and “can’t step off curb to get into car – need assistance,” among others. In addition, in the case of walking impairments that have advanced to needing to use hand/arms for support, numerous other activities can be envisioned to be impacted: housework, home-maintenance, gardening, sports (numerous mentioned), traveling, and “walking while carrying infant.”

**Table 3:** Patient-reported activities impacted by ataxia: selection of verbatim reports from online survey from questions 2 and 3 (derived from *N* = 147 patient respondents)

Concept measured	Patient-reported activities impacted by ataxia		Frequency of report [high (H, medium (M), low (L)]
*Evaluation of gross motor functions*			
Gait and walking	Walking; walking alone; walking for exercise or pleasure; walking slow Walking in crowds In a wheelchair, cannot walk Walking longer than 15 min; longer than 2 mi Cannot walk over rocks, grass, beach, uneven surfaces, slick surfaces; countryside walking; walking into places with elevated ramp Walking at night Walking—need railing Walking dog difficulty *Getting on/off airplane; traveling; cannot travel alone*	*Going by myself to store*; need shopping cart to walk straight Walking carrying hot food (food service industry); *carrying pot of water to stove; open cups;* walking while carrying infant Cannot run; *sports* (*numerous varieties of sports mentioned)*; *riding a bike*; pools need handicap entrance, or *I cannot swim* Problems walking on stairs; climbing stairs without a railing Cannot step off curb to get into car—need assistance stepping over curbs Cannot carry things that don't fit in my basket on walker; *carrying things*; *large or heavy objects; furniture;* getting mail *Housework; home maintenance; gardening*	H
Stance and balance *(while standing still)*	Standing up; standing in shower; free standing; standing in choir; standing in line Standing with feet close together; cannot stand or walk in tandem Standing and holding babies; hug babies Socializing—standing while holding a drink Standing longer than 1 min by myself; more than 10 min no longer possible Cannot step off curb to get into car—need a hand or arm	*Putting food in the oven* Cannot use the stove or oven because they don't have an automatic shut-off (in case I fall when I'm cooking) *Housework; home maintenance; dizziness impacts gardening* *Sports* (*numerous varieties of sports mentioned)* Balance issues forced me to give up motorcycle riding Balancing on one foot Standing on tiptoes to reach something high	H
Quality of Sitting Position	Sitting *Bathroom*	Squatting down without falling over *Driving*	M
*Evaluation of physical functioning, targeted systems*^*1*^			
Speech	Speaking clearly to people; *avoid talking a lot* *Socializing*	Singing *Talk on phone* *Playing with grandchildren*	M
Swallowing	Swallowing Drinking	Eating Eat slowly because of choking	L
Oculomotor	Reading Dizziness *Shaving*	Computer work *Driving*; driving at night; I had to stop driving because of the vertigo *Sports* (*numerous varieties of sports mentioned)*; *shooting guns*	H^2^
*Evaluation of upper limb functioning*			
Finger movement and target accuracy^3^ Hand Tremor^3^	*Shaving* Using computer mouse; dialing phone; texting Sewing; needle craft; handling power tools *Car repairs; home repairs* *Bathroom*	*Dressing* *Driving* *Food preparation*; cannot serve self food *play instrument (several mentioned)* *Sports (several mentioned)*	H
Fine motor accuracy	Drawing Writing; writing illegible; only *understand signature* Typing; using keyboard	*Turn key in lock* tape and so many small motor movements needed for all types of office work and paperwork Sewing; needle craft *Gave up woodworking; power tools; mechanical work*	H
Alternating hand movements	Brushing teeth *Turn key in lock* Knife skills; *preparing food* Unable to turn pages, *straightening papers*; *tape and so many small motor movements needed for all types of office work and paperwork* *Gave up woodworking; power tools; mechanical work*	*Opening heavy door* *Cannot lift or carry big things; heavy things; great-nephew* *Sports (numerous mentioned)* *Bathroom* *Driving*	H
*Evaluation of lower limb functioning*			
Lower limb assessment^4^	Driving *See walking and gait responses*	Putting on shoes *See stance and balance responses*	H
***Evaluation of activities of daily living (ADLs)***			
Cutting food and feeding self	Poor control while cutting food Knife skills; food prep	*Keeping firm grasp on items* Eating	M
Dressing	Dressing Fastening buttons; fumble with zippers and buttons	Belts Putting on shoes; tying shoes	L
Personal hygiene	Personal hygiene Standing in shower; showering Squatting down without falling over Bathroom	Applying makeup *Poor control of scissors* Brushing teeth; very poor coordination in flossing my teeth *Fine motor skills; keeping firm grasp on items; opening doors and tops of things*	M
Falling	*See walking and gait responses*	*See stance and balance responses*	N/A

*Italicized responses* reflect reports that may be more tenuously associated with the stated concept, as that the relationship may be less clear. ^1^No activities reported reflecting challenges with coughing or bladder function. ^2^While there are many reports of driving impacts, they are non-specific to the reason why the respondent cannot drive; the frequency of M reflects that uncertainty. ^3^The mapping showed the same list of impacted activities for these 2 concepts: finger movement and target accuracy and hand tremor; reporting condensed in table to reduce duplication. ^4^Impacted activities related to lower limb functioning and control are proposed to also include that where lower limb performance is paramount, e.g., walking and gait and stance and balance

Reports of difficulty with stance and balance in a non-gait context included “standing in shower,” “standing on tiptoes to reach something high,” “standing longer than 1 min by myself,” and standing more than 10 min (is) no longer possible;” additional examples include “can’t bend over safely because of wobble,” “squatting down without falling over,” and the ability to participate in numerous sports or exercise activities.

Considering the complex task of dressing as included in the FARS-ADL domain, mentions of “dressing” or “fumble with zippers and buttons” in the activities question directly support the importance to assessing this day-to-day activity or ability.

General broad concept activities were also reported that do not map to a single concept, but may explore a range of abilities, compensations, and losses: “I am still able to do most activities. I just need more time to do them,” “need my husband’s assistance with daily chores and grandchildren childcare,” “retired early,” “getting employment,” “work part-time,” “playing with grandchildren,” “limited social activities,” and “visiting friends.”

In general, relative-reported symptoms and impacts revealed similar themes as those reported by the patients; however, they provided additional insights not seen in patient reports (Table 4). Detailed statements include the following: “Cannot toilet unassisted,” “transferring in and out of bed,” “takes all energy to walk with assisted devices,” “bending down to open the oven,” “unable to cross streets – fear of being unable to move fast enough to get out of way of oncoming auto,” and “fear of falling.” Several reports mentioned difficulty with working for pay and community work: “retired early,” “going to work (getting ready, being punctual, feeling well enough),” “cannot volunteer,” and difficulty with “helping parents.” Several relative reports spoke to general impacts of the disease: “all aspects of normalcy,” impacts “anything that requires strength or coordination or endurance or balance,” and “most daily activities (physical) can still be accomplished if they are simple and routine.” Also included in the relative reports were statements about loss of patient independence, “unable to live and function on his own.”

**Table 4:** Supplemental reports of symptoms or impacts of ataxia: selection of verbatim reports from relative (derived from *N* = 80 respondents)

Concept measured	Additional patient symptoms or impacts of ataxia, as reported by relatives^1^
*Evaluation of gross motor functions*	
Gait and walking	Cannot negotiate walking around corner—shoulder will hit wall; falls to the left when walking Clumsy (bumps into things) Falls to the left when walking Walks like Frankenstein when cold Balance in walking sometimes worse than other times, especially later in the day Trouble walking down stairs/hills Has limited energy and mobility so even something simple like shopping has been drastically affected Unable to cross streets—fear of being unable to move fast enough to get out of the way of oncoming auto (Impacts) walking out to sports fields to watch grandchild's game (Impacts) walking and holding hands Takes all energy to walk with assisted devices Cannot go to stadiums or anyplace with steps; walking up bleacher steps
Stance and balance *(while standing still)*	Will fall if stands with eyes closed; falls backwards when standing too long or when eyes closed; anxiety due to falling Standing for short periods of time even to cook or to travel tires her; (impacts) cooking unattended Takes a while for him to get balanced when rising out of chair (impacts) standing on ladder to fix thing Cannot pick up items that have dropped to floor Bending down to open the oven Does not go shopping by herself as getting out of car and finding a cart is impossible
Quality of sitting position	Cannot toilet unassisted; sometimes unable to go to the bathroom by herself Transferring in and out of bed Getting up and down off the ground Gets dizzy when going from lying to sitting (but does fine with sitting to standing)
*Evaluation of physical functioning, targeted systems*	
Speech	Slurred speech when tired
Oculomotor	Riding in car is challenging because causes dizziness- motion sickness
*Evaluation of upper limb functioning*	
Finger movement and target accuracy	Using a smart phone Putting away dishes Riding in car is challenging because causes dizziness- motion sickness
Hand tremor	Cooking (tremor in hands)
Alternating hand movements	Cannot carry anything Handing item off to another person Balancing items while walking Holding book is a challenge Dining with smaller groups in public
*Evaluation of activities of daily living (ADLs)*	
Cutting food and feeding self	Cut food at dinner; cutting meat Food preparation Feeding himself Feed and care for grandchild
Personal hygiene	Takes a while for him to get balanced when rising out of chair Cannot toilet unassisted; sometimes unable to go to the bathroom by herself
Falling	Anxiety due to falling Falls backwards when standing too long or when eyes closed

^1^As the concepts reported by patients and relatives were consistent, several categories did not yield additional insights: swallowing, bladder, cough, fine motor accuracy, lower limb, and dressing

Lastly, examination of the PROM-Ataxia patient focus group discussion provided further insights into symptoms and impacts of disease (Table 5). While a range of walking surfaces were reported as impacted by the survey (e.g., cannot walk over rocks, grass, beach, uneven surfaces, slick surfaces; countryside walking; walking into places with elevated ramp), the focus group additionally identified that walking on flat surfaces and hills was challenging. While the survey mentioned stairs, the focus group supported more complicated maneuvers, such as the use of stepstools. While the concept of “travel” was mentioned in the survey, aspects of “using public transportation without assistance (bus, train, airplane)” were endorsed by the focus group.

**Table 5:** Ataxia patient focus group: Select insights from PROM-Ataxia development towards patient experience

Concept measured	Additional patient symptoms or impacts of ataxia, as learned from the ataxia patient focus groups, and featured in the PROM-Ataxia measure
*Evaluation of gross motor functions*	
Gait and walking	Additional probes of walking on flat surfaces and hills Additional probes of standing on stepstool Additional probes of carrying packages Probes the ability to catch myself and prevent fall when I stumble Probes moving about the house without assistance Probes getting in and out of a car Probes using public transportation without assistance (bus, train, airplane)
Stance and balance *(while standing still)*	Probes unsteady standing/walking on flat surfaces Probe of lose my balance and fall Probe of I can bend down and pick something off the floor without help Probes getting on and off toilet without assistance Probes getting up off the floor without help
Quality of sitting position	Probes getting on and off toilet without assistance Probes rolling over in bed Probes getting in and out of bed without assistance
*Evaluation of activities of daily living (ADLs)*	
Dressing	Adds more details of dressing: socks, earrings, watch, belt
Personal hygiene	Probes get on and off toilet without assistance
Falling	Has question on catch myself and prevent fall when I stumble

There were several more complex items incorporated in the PROM-Ataxia scale and endorsed by the focus group that may reflect impacted abilities. “Getting on and off toilet without assistance” taps many potential symptoms of stance, balance, or sitting skills; it is also a component of the personal hygiene FARS-ADL item. Similarly, the item of “catch myself and prevent a fall when I stumble” is related to gait/walking as well as the falling item on the FARS-ADL.

## Discussion

Using the exhaustive and novel concept elicitation dataset that served as the basis for the development of the PROM-Ataxia, the first patient outcome measure derived from and for use specifically in the ataxia patient population, we report for the first time that the four observer/clinician administered COAs indeed are deep and successful probes of patient experience of the cerebellar motor ataxia syndrome. Using the patient voice directly through open-ended survey questions and the ataxia patient focus groups, this study demonstrates linkage between patient-reported symptoms and the concepts measured in the clinical assessments. Whereas previous studies have focused on the psychometric properties of these measures in ataxia populations [23], few have examined the patient centricity of these measures explicitly [43]. Herein, we critically evaluate this as driven by the need to demonstrate patient-relevance of the COAs [14].

We found that, with the exception of cough, each concept evaluated in the 4 COAs measured a patient-reported symptom of cerebellar ataxia and had multiple reports of impacts on patient function to inform the meaningfulness of the task on the patient’s day-to-day living. In particular, “gait and walking” and “stance and balance” were frequently reported symptoms of disease and were associated with numerous impacted activities. Similarly, items evaluating hand/arm movements or coordination, including both gross-motor and fine-motor movements, mapped onto frequently reported symptoms and disease impacts. Further, concepts involving patients’ ability to carry out their daily activities were shown to be highly relevant, such as the 3 items on the FARS-ADL.

The five cardinal components of the cerebellar motor examination—gait and balance, upper limb coordination, lower limb coordinator, speech clarity, and oculomotor control—are core to many aspects of movement, physical functioning, and activities of daily living. Consequently, responses reflecting symptoms or impacts due to balance and coordination challenges were mapped to multiple concepts. This is exemplified by the activity of “driving” which can be associated with any or all of the following concepts: oculomotor impairment (challenges with vision or depth perception), impaired upper limb functioning (control of steering wheel), impaired lower limb functioning (control over gas pedal), or stance/balance (getting in and out of the car). The impacted activity of “home repairs” may be linked to any or all the following concepts: tremors (holding or positioning the tool), finger movement and target accuracy (working with the tool), walking and gait (carrying tools or supplies to place of work), or oculomotor (lack of depth perception, blurry vision).

Our novel data therefore reveal for the first time that there is a complex and interactive mosaic mapping of COA items in the observer-derived scales with the array of subjective and meaningful reports by patients/families of the lived experience with the disease. Each item of the COA maps to multiple real-life manifestations; many real-life impacts of disease map to multiple individual items on the COAs. These results emphasize the inadvisability of attempting to single out any one item on the COAs as indicative of the totality of the patient’s lived experience. Our data underscore the complexity of cerebellar ataxia manifestations and how they are accurately reflected in sets of items on the COAs, which were conceptualized and developed by international consortia of knowledgeable ataxia experts over the past 3 decades, drawing on the deep clinical knowledge of motor ataxiology stretching back to the late 1800s.

We note that the relatives’ survey responses echoed similar themes present in the patient responses, but they also provided additional insights such as those relating to using the bathroom or being able to work. It may be that relatives are more likely to report the impacts that are of a more personal nature.

There were fewer reports of impacts on abilities to self-care (shower, brush teeth, brush hair), prepare food for self or family, and use the toilet independently. These abilities are commonly regarded as being important to patients and caregivers and were expected to be reported with higher frequency. It is possible that the broad nature of the questions did not readily elicit these types of responses. Another factor may be that more personal activities are less spontaneously reported in open-ended surveys. Further work may help clarify this observation.

Patients reported gait impairment across a continuum of severity in this cross-sectional study. This ranged from slow walking to wall walking to keep balance, stumbling, falling, inability to walk and carry anything, inability to climb stairs or go down curbs, and finally confined to wheelchair. This gradation of gait disintegration, a known feature of the ataxia journey in neurodegenerative disorders, maps onto the walking/gait, and balance/stance concepts captured in stepwise fashion in the COAs. Similarly, the evolution of dysarthria maps well onto the continuum of the item scoring the degree of speech impairment, the dissolution of articulatory clarity progressing from mild slowing, and tripping on syllables, to progressive inability to make oneself understood on the telephone and in person, to the point of unintelligible speech (Fig. 1, Table 6). It may therefore be expected that the data from this cross-sectional cohort could inform meaningful changes to an individual patient over time, but longitudinal studies to test this hypothesis are pending.

**Table 6:** Placement of survey responses on a hypothetical disease continuum for cerebellar ataxia

	Initial impairments						Maximal impairments
Gait/walking	Slow walking Unable to cross streets—fear of…unable to move fast enough…oncoming auto	Walking in crowds Cannot turn or stop quickly	Wall walking to keep balance	Stumbling Challenges walking on uneven surfaces Falling	Cannot walk and carry anything Cannot walk without my walker	Cannot climb stairs or go down curbs	Wheelchair bound
Balance	Difficulty regaining balance	Standing with feet close together Cannot stand…in tandem	Standing to hug/hold baby	Getting balanced when rising from a chair	Putting food in the oven Bending down to open oven	Standing for short periods of time even to cook	Cannot stand
Speech	Slow speech	Slurred speech	Poor speech	Talking on phone	Difficulty with social conversations	Avoid talking a lot	{can’t speak}^1^

All responses are taken from the original survey data set, reflecting either symptoms or activities, reported by the patient or relative (except *{can’t speak}*). The granularity of the data did not allow for this exercise with the upper limb functioning assessments. ^1^{can’t speak} was not reported in survey, included in this diagram to illustrate highest possible impairment

Because the measures were examined at the item-level, they can be used to evaluate current and future modifications of existing scales or the development of new scales that include these items or concepts. This could apply for example to the f-SARA, a modification of the existing SARA, which is composed of the first 4 items of the SARA. The present analysis could be used to evaluate the patient-centeredness of the f-SARA to understand how the items translate to meaningful symptoms and aspects of day-to-day living.

These data are cross-sectional, using the patient voice to draw attention to those aspects of the disease that are particularly meaningful to patients, and relating these concepts to the COAs. Our study therefore provides new insights into the relevance of the concepts evaluated in the COAs. Further research is needed to build on these observations and evaluate longitudinal relationships between changes in COA scores and meaningful impacts on patients’ lives.

## Limitations

In some instances, the data generated by patients and families needed interpretation. For example, there were instances where patient-reported symptoms affected activities and vice versa. In order to structure the tables and analyze the collected data, survey responses were interpreted using a blend of clinical familiarity with the cerebellar ataxia population and COA development fundamentals and best-practices [15, 16]. The survey was administered online and in English, exploring the perspectives of respondents who can navigate the technology and speak the language. While the survey was administered to both patients and relatives, any potential relationship between specific relative and patient respondents is unknown. Further, while the scope of this work focused on scales primarily assessing ataxia symptoms and impacts, it is noted that non-ataxia symptoms such as those evaluated in the Inventory of Non-Ataxia Signs (INAS) may contribute meaningfully to patient experience of their disease. While progressive cerebellar ataxias have similar core characteristics, there is great heterogeneity in clinical manifestations and progression of symptoms among the various disorders that must be taken into consideration. Lastly, there were several symptoms and impacts reported in the survey and focus groups that were not addressed by the selected COAs and were not described in this analysis. These include emotional impacts, cognitive impairment, pain, neuropathy, and sleep issues. The broader presentation of the totality of impacted domains can be found in Schmahmann et al. [21].

## Conclusions

There is a complex and interactive mosaic mapping of COA items in the observer-derived ataxia rating scales with the subjective reports by ataxia patients/families of the relevance of these COA items to their lived experience. Each item of the COA maps to multiple real-life manifestations; each real-life impact of disease maps to multiple individual items on the COAs. This work therefore anchors the commonly used ataxia COAs in the symptoms and experience of patients and families with ataxia. Our data confirm the content validity of these measures and enhance the understanding of the impact of disease on patients’ daily life and care by demonstrating the searching relevance of the COAs. These findings have the potential to enrich patient-clinician conversations focusing on developing meaningful and personalized treatment goals. The methodology of this study is both novel in its approach and in the uniqueness of the data available for analysis because it was derived from the previously unpublished but publicly available dataset used as the basis for the concept elicitation of the PROM-Ataxia. The approach used in our investigation may serve as a model for similar COA-patient experience validation studies in other diseases.

## Abbreviations

ADL: Activities of daily living
BARS: Brief Ataxia Rating Scale
COA: Clinical outcomes assessment
FARS: Friedreich’s Ataxia Rating Scale
FARS-ADL: Activities of daily living domain of the Friedreich’s Ataxia Rating Scale
f-SARA: Modified functional Scale for the Assessment and Rating of Ataxia
ICARS: International Cooperative Ataxia Rating Scale
INAS: Inventory of non-ataxia signs
MSA-C: Multiple system atrophy cerebellar type
PROM-Ataxia: Patient Reported Outcome Measure of Ataxia
SARA: Scale for the Assessment and Rating of Ataxia
SCA: Spinocerebellar ataxia
